# 'We Had More in Common Than I Thought': Scamming as an Undergraduate Service Learning Topic Involving Older Adults

**DOI:** 10.1093/geroni/igab046.2795

**Published:** 2021-12-17

**Authors:** Marjorie Getz

**Affiliations:** Methodist College, Peoria, Illinois, United States

## Abstract

An ongoing activity that cuts across several courses in the Gerontology Certificate Program at our College is the completion of implicit association exercises focused on age. Most college students show a distinct preference for those who are younger adults. It is difficult to get across to these students that the construct of being an adult is appropriate for all people beyond adolescence without relevance to age. College students enrolled in healthcare programs often have distorted views of aging and may not fully appreciate that all adults may share common aspects of their current lives. We describe qualitative analyses of reflections taken from an undergraduate psychology course that included a service learning component involving older adult learners. The service learning lessons focused on victimization associated with fraud and scamming. The classroom structure involved round table discussions with direct contact between college students, older adults and local law enforcement personnel. Reflective practices were used to integrate course content (development in adulthood) into this service learning activity. We report on qualitative data taken from student reflections. Content analyses of reflective essays identified five themes which operated to produce stronger identification between age groups: frequency of being scammed across all 21 participants; insight that learning continues across the lifespan; understanding that broad learning challenges impact people (for different reasons) at both ends of the adult age spectrum; respect for adoption of strategies that facilitate learning/compensate for cognitive changes that occur with aging; acknowledgement that familiarity breaks down barriers between people.

